# Laparoscopic surgery for inguinal hernia: Current status and controversies

**DOI:** 10.4103/0972-9941.27735

**Published:** 2006-09

**Authors:** Deepraj S Bhandarkar, Manu Shankar, Tehemton E Udwadia

**Affiliations:** Department of Minimal Access Surgery, P. D. Hinduja National Hospital and Medical Research Centre, Mumbai, India

**Keywords:** Inguinal hernia, laparoscopic repair, open repair, totally extraperitoneal, transabdominal preperitoneal

## Abstract

Repair of inguinal hernia is one of the commonest operations performed by surgeons around the world. The treatment of this common problem has seen an evolution from the pure tissue repairs to the prosthetic repairs and in the recent past to laparoscopic repair. The fact that so many hernia repairs are practiced is a testimony to the fact that probably none is distinctly superior to the other. This review assesses the current status of surgery for repair of inguinal hernia and examines the various controversial issues surrounding the subject.

The subject of repair of inguinal hernia has been full of controversy ever since Eduardo Bassini of Padua University described his method of repair in the manuscript ‘Radical Cure of Inguinal Hernias’ way back in 1887. The fact that more than a hundred repairs have been described for inguinal hernia and practiced at some time or the other over the past century are a testimony to the fact that none has been considered distinctly superior to the others. In recent years, however, the use of mesh for repair of inguinal hernia has become a norm. Reduction in the recurrence rate from more than 15% with tissue repairs to less than 1%, reduction in the postoperative pain and a shorter convalescence have all contributed to the popularity and widespread use of the tension-free mesh repairs. The laparoscopic repair of inguinal hernia, a relatively newer modality in the armamentarium of the surgeon, has been around only for a little over a decade. Although perhaps not practiced as widely as laparoscopic cholecystectomy is for gallstone disease, laparoscopic repair of inguinal hernia has established its rightful place in the surgical practice. This review assesses the current status of groin hernia surgery (for repair of inguinal hernia in adults) and examines the various controversial issues surrounding the subject.

## Evolution of laparoscopic hernia repair

Nyhus and Stoppa developed the concept of preperitoneal repair of inguinal hernia in an effort to reduce the high recurrence rates associated with the anterior repairs popular around that time - most of which in fact were tissue, as against, prosthetic repairs. Around the same time, Lichtenstein[1] and others started performing anterior tension-free mesh repairs, also in an attempt to reduce the recurrence rate, postoperative pain and long convalescence associated with traditionally performed Bassini, Shouldice and McVay repairs. Ironically, Lichtenstein's concept of tension-free repair by routine implantation of a mesh, which was scathingly criticized as late as 1990,[2] is today considered the ‘gold standard’ in the open treatment of groin hernias.

The laparoscopic repair of inguinal hernia was introduced in early 1990s. Unfortunately, the early attempts included procedures such as intraperitoneal onlay mesh repair and use of a small patches and plugs. These methods, which were in sharp contradiction to the well-founded principles of open tension-free repairs, resulted in an alarmingly high recurrence rates and were instant failures. These were soon abandoned in favor of the laparoscopic approaches emulating the ‘unilateral Stoppa’ or the Wantz repair.[3] In this type of repair, a sheet of mesh is placed in the preperitoneal space to cover the myopectineal orifice of Fruchaud and is fixed with sutures to prevent its migration. In the laparoscopic repair of groin hernia, the placement of the mesh may be accomplished either via the transperitoneal route (transabdominal preperitoneal repair - TAPP) or the extraperitoneal route (totally extraperitoneal repair - TEP; the mesh is fixed in place with fixation devices such as tacks or with sutures).

## LAPAROSCOPIC VERSUS OPEN (NON-MESH) REPAIR OF INGUINAL HERNIA

A recent meta-analysis of randomized controlled trials compared the results of the laparoscopic technique (TAPP and TEP) with the results of the Shouldice and other open non-mesh techniques of repair of inguinal hernia.[4] A total of 27 studies were identified and formed the basis of this meta-analysis. The following section deals with the various aspects of laparoscopic and open non-mesh repairs compared in that meta-analysis.

### Operating time

The average operative time for TAPP/TEP was 68 min versus 54 min for the Shouldice repair. A comparison with the other non-mesh techniques also showed a significantly longer operating time for laparoscopic procedures: 61 versus 43 min.

### Total morbidity

The total morbidity was significantly lower after laparoscopic operations (13.9%) in comparison to the Shouldice repair (16.5%) The morbidity rates between laparoscopic repair and open non-Shouldice suture repair were not significantly different: 21.2% versus 19.0%.

### Serious intraoperative complications

There were no serious bowel injuries in the analyzed trials. However, there were two (0.07%) bladder injuries (both during TAPP) and one urinary bladder lesion after a Shouldice repair (0.03%); these were not statistically significant. Of all the analyzed series, one study reported bleeding from inferior mesenteric vessel during a TAPP operation. No cases of vascular injury were reported in open operations.

### Wound infection

Although the wound infection rate was not significantly different between laparoscopic techniques (0.5%) and the Shouldice repair (0.9%), comparison with the non-Shouldice suture repair (1.2%) showed that the rate of wound infection was significantly lower after laparoscopic techniques (0.6%).

### Hematoma

The meta-analysis of hematoma after TAPP/TEP showed an incidence of 4.2% as against 6.4% after Shouldice repair. Comparison of the laparoscopic techniques with other open non-mesh techniques showed no significant difference in the incidence of hematoma.

### Seroma

Not all studies included in the meta-analysis defined and evaluated seroma as a complication. But the studies that did evaluate this parameter showed that the incidence of seroma was significantly higher after laparoscopic (4.4%) than after Shouldice repair (1.2%) or after other open non-mesh techniques (0.5%).

### Urinary retention

In few of the series in which the incidence of postoperative urinary retention was examined, there was no difference between the laparoscopic and open non-mesh techniques, neither between laparoscopic techniques and Shouldice repair.

### Time to return to work

The mean time for return to normal activity after Shouldice repair was 31.2 days and after laparoscopic repair it was 21.2 days. Also, the convalescence was significantly shorter after laparoscopic (14.6 days) as compared to non-Shouldice techniques (22.3 days).

### Inguinal paresthesia

The incidence of inguinal paresthesia was significantly lower at 0.8% after laparoscopic operations as compared to 5.0% after Shouldice repair. Similarly, the studies comparing laparoscopic with open non-Shouldice techniques also reported significantly fewer nerve lesions after laparoscopic operations.

### Chronic pain

The incidence of chronic pain in the inguinal area was significantly lower after laparoscopic techniques (2.2%) as against after Shouldice repair (5.4%). A comparison of the endoscopic with the open non-Shouldice suture techniques (9%) also showed a lower incidence of chronic pain after laparoscopic repair (3.9%).

### Testicular atrophy

No significant difference was found in the reported incidence of testicular atrophy between the laparoscopic techniques and the Shouldice repair or the non-Shouldice repairs.

### Hernia recurrence

Analyzed data from the studies included did not show any difference in the recurrence rate between the laparoscopic techniques (1.6%) and the Shouldice repair (2%). However, the recurrence rate following open non-Shouldice suture techniques was significantly higher at 5.4% as compared to the 3% for laparoscopic repair.

Table 1 and 2 summarize the results of the meta-analysis and show the differences found in a comparison of the laparoscopic techniques with the Shouldice repair and the other open non-Shouldice suture techniques for the various parameters.

**Table 1:** TAPP/TEP versus Shouldice repair

Advantage for TAPP/TEP	Advantage for Shouldice	No difference (*P*>0.05)
↓ Total morbidity	↓ Operation time	Wound infection
↓ Hematoma	↓ Seroma	Urinary retention
Faster return to work		Bowel injury
↓ Nerve injury		Bladder injury
↓ Chronic groin pain		Vascular injury
		Testicular atrophy
		Recurrence rate

(Adapted from Bittner R *et al*[4]), TAPP- Transabdominal preperitoneal repair, TEP - Totally extraperitoneal repair

**Table 2:** TAPP/TEP versus open non-Shouldice repair

Advantage for TAPP/TEP	Advantage for open non-Shouldice repair	No difference (*P*>0.05)
↓ Wound infection	↓ Operation time	Total morbidity
Faster return to work	↓ Seroma	Hematoma
↓ Nerve injury		Urinary retention
↓ Chronic groin pain		Bowel injury
↓ Recurrence rate		Bladder injury
		Vascular injury
		Testicular atrophy

(Adapted from Bittner R *et al*[4]), TAPP- Transabdominal preperitoneal repair, TEP - Totally extraperitoneal repair

The authors submitted that they were unable to adequately analyze parameters such as the learning curve for the individual operation techniques, the incidence of injury to the vas deferens, the incidence of missed contralateral hernias and the incidence of trocar-site hernias.

An important point to keep in mind when interpreting the results from the series included in this meta-analysis is that all studies were conducted in centers of excellence by surgeons with a special interest in laparoscopic hernia repair. These data cannot be extrapolated to surgical practice across the world, as these results may not be reproducible in a cross-section of hospitals. Also, as the laparoscopic procedures are techniques that necessitate implantation of mesh, they are not strictly comparable to the open non-mesh suture techniques in all respects.

## LAPAROSCOPIC VERSUS OPEN (MESH) REPAIR OF INGUINAL HERNIA

The same group had previously published a meta-analysis of randomized controlled trials comparing the results of the laparoscopic technique (TAPP and TEP) with the results of the Lichtenstein and other open mesh techniques of repair of inguinal hernia.[5] A total of 34 studies formed the basis of this meta-analysis. The following section deals with various aspects of laparoscopic and open mesh repairs compared in that meta-analysis.

### Operating time

The average time taken for TAPP/TEP (65.7 min) was significantly longer than that for the Lichtenstein repair (55.5 min). A comparison with the non-Lichtenstein open mesh techniques also showed that the operating times were significantly longer for the laparoscopic operations.

### Total morbidity

The meta-analysis of total morbidity indicated that there was no difference between the laparoscopic techniques (28.4%) and the Lichtenstein repair (28.3%). However, this result was strongly influenced by the veterans affairs multicenter trial (VAMT) that showed a rather high morbidity in the laparoscopic group.[6] Upon exclusion of the VAMT data, however, the total morbidity was found to be significantly higher for the Lichtenstein repair. The meta-analysis of studies comparing endoscopic repair with other open mesh techniques revealed that morbidity is significantly lower after laparoscopic repair (29.9% versus 24.6%).

### Serious intraoperative complications

In the studies summarized, there were four (0.1%) intraoperative bowel injuries during laparoscopic repairs and two (0.06%) during open mesh repairs. There was no statistically significant difference between the two groups. The incidence of urinary bladder injuries in laparoscopic repairs was significantly higher at 0.1% versus zero after open mesh repairs. Also, the overall incidence of vascular injury during laparoscopic repairs was 0.09% as against no reported cases during open operations.

### Wound infection

The wound infection rates were significantly lower after laparoscopic techniques (1%) than after the Lichtenstein operation (2.7%) and other open mesh repairs (2.4%).

### Hematoma

The incidence of inguinal hematoma was found to be significantly lower after the laparoscopic repairs (13.1%) than after the Lichtenstein repair (16.0%) as well as with the other open mesh techniques (14.3%).

### Seroma

Only some of the analyzed studies defined and evaluated seroma as a complication. A significantly higher incidence of seroma was found after laparoscopic procedures (12.2%) in comparison to the Lichtenstein repair (8.9%). There was no statistically significant difference in the postoperative incidence of seroma between laparoscopic repair and non-Lichtenstein open mesh repairs.

### Urinary retention

The incidence of postoperative urinary retention was no different after laparoscopic and Lichtenstein repairs.

### Time to return to work

The mean time to return to work or normal activities was 14.8 days after laparoscopic operation as compared to 21.4 days after Lichtenstein repair. In addition, as compared to the non-Lichtenstein open mesh techniques, the convalescence was significantly shorter after the laparoscopic repairs.

### Inguinal paresthesia

The studies that included information about inguinal or scrotal paresthesia showed that the laparoscopic operations resulted in a lower incidence (0.46%) of paresthesia in comparison with the Lichtenstein repair (3.9%). The studies comparing laparoscopic with open non-Lichtenstein mesh techniques also reported significantly fewer symptoms of nerve lesions after laparoscopic operations.

### Chronic pain

A significantly lower incidence of chronic pain was documented after laparoscopic operation (7.6%) as compared to the Lichtenstein repair (12.7%).

### Testicular problems

There was no significant difference between laparoscopic and open mesh repair in the incidence of postoperative testicular problems.

### Hernia recurrence

A summary of the data from included studies showed a significantly higher recurrence rate after laparoscopic techniques (5.5%) than after Lichtenstein repair (2.7%) Once again, these data were strongly influenced by the poor results in the laparoscopic group from the VAMT.[6] When these data are excluded, there was no statistical significant difference between laparoscopic and Lichtenstein repair in the recurrence rate. When comparing the laparoscopic techniques with open non-Lichtenstein mesh repairs, there was no significant difference in hernia recurrence.

Table 3 and 4 summarize the results of the meta-analysis and show the differences found in a comparison of the laparoscopic techniques with the Lichtenstein repair and the other open mesh repairs for the various parameters.

**Table 3:** TAPP/TEP versus Lichtenstein repair

Advantage for TAPP/TEP	Advantage for Lichtenstein	No difference
↓ Wound infection	↓ Operating time	Total morbidity
↓ Hematoma	↓ Seroma	Bowel injury
Faster return to work	↓ Hernia recurrence	Urinary bladder lesion
↓ Nerve injury		Vascular injury
↓ Chronic groin pain		Urinary retention
		Testicular problems

(Adapted from Schmedt CG *et al*[5]), TAPP- Transabdominal preperitoneal repair, TEP - Totally extraperitoneal repair

**Table 4:** TAPP/TEP versus open non-Lichtenstein mesh repair

Advantage for TAPP/TEP	Advantage for open non-Lichtenstein mesh	No difference
↓ Total morbidity	↓ Operating time	Bowel injury
↓ Wound infection	↓ Urinary bladder injury	Vascular injury
↓ Hematoma		Urinary retention
Faster return to work		Seroma
↓ Nerve injury		Chronic groin pain
		Testicular problems
		Hernia recurrence

(Adapted from Schmedt CG *et al*[5]), TAPP- Transabdominal preperitoneal repair, TEP - Totally extraperitoneal repair

This meta-analysis showed that the laparoscopic techniques carry definite advantages over the open mesh repairs in terms of local complications and pain-associated parameters. Except for the total morbidity, seroma formation, chronic inguinal pain and recurrence rate, there were no major differences in the results between the Lichtenstein repair and the other open mesh techniques. In this meta-analysis, several of the trials included patients operated upon by surgeons with limited experience with laparoscopic hernia repair and hence some of the data relating to intraoperative bladder and vascular injury as well as recurrence rates are likely to be skewed.

## TAPP OR TEP FOR INGUINAL HERNIA REPAIR?

The choice of approach to the laparoscopic repair of inguinal hernia is controversial. There is a scarcity of data comparing the TAPP approach with the TEP approach and questions remain about their relative merits and risks. A recently published Cochrane review compared the clinical effectiveness and relative efficiency of TAPP and TEP.[7] All published and unpublished randomized controlled trials and quasi-randomized controlled trials comparing TAPP with TEP as well as non-randomized prospective studies were included in this review.

The Cochrane search identified only one prospective randomized trial that fulfilled all the criteria for inclusion in the review.[8] This study reported no statistical difference between TAPP and TEP when considering duration of operation, hematoma, length of stay, time to return to usual activity and recurrence. The eight non-randomized studies also reviewed suggested that TAPP is associated with higher rates of port-site hernias and visceral injuries whilst there appeared to be more conversions with TEP. Vascular injuries and deep/mesh infections were rare and there was no obvious difference between the groups. Unfortunately, none of the series reported economic / cost-benefit analysis. Very limited data were available on learning effects but these data suggest that operators become experienced after about 30 to 100 procedures. It appears that currently available data do not allow firm conclusions to be drawn regarding the superiority of one approach over the other and prospectively randomized studies with adequate number of patients in each arm are necessary to answer this question.

## FIXATION OF THE MESH VERSUS NO FIXATION

Felix *et al*[9] in a multicentric retrospective review examined the factors beyond the learning curve that could lead to recurrence after laparoscopic hernia repair. Of the 10,053 hernias repaired by TAPP or TEP, 35 patients developed a recurrence. Inadequate lateral or medial fixation was found to be a cause of recurrence in 19 of these cases. Thus, adequate fixation of the mesh has been always considered an important factor in reducing the likelihood of recurrence.

Two prospective randomized studies have examined the issue of fixation of the mesh - one in TAPP and the other in TEP. Smith *et al*[10] randomized 502 consecutive patients undergoing elective TAPP to having stapled or non-stapled hernia repairs. A total of 263 non-stapled and 273 stapled repairs were performed and the median follow-up was 16 months. There was no statistical difference in the incidence of recurrence (0 out of 263 in non-stapled group, 3 out of 273 in stapled group). Also, there was no significant difference in the operative time, port-site hernia and chronic groin pain between the two groups. The authors concluded that it was not necessary to secure the mesh during laparoscopic TAPP inguinal hernia repair. Similarly, Ferzli *et al* undertook a prospective randomized study to assess the necessity of stapling in 100 TEPs in 92 patients.[11] There were no complications in either group and no recurrences at the end of 12 months The authors pointed out that in the US, hernia repairs performed without staples translated into a cost saving of $120 per patient.

The placement of mesh in the preperitoneal space without fixation, however, needs to be put in proper perspective. If fixation is to be omitted, it is absolutely mandatory that a wide enough preperitoneal space be created to accommodate a mesh no smaller than 15 × 12 cm in size. The mesh should overlap the edges of the apparent as well as the sites of all potential defects by at least 3–4 cm on all sides. Also, the mesh should neither be slit nor allowed to fold up in the preperitoneal space during release of pneumoperitoneum / pneumopreperitoneum. Also, in large hernias, particularly of the direct variety, it may be prudent to obtain an adequate overlap around the edges of the defect as well as fix the mesh at least two points superiorly to prevent migration of the mesh.

## ANESTHESIA FOR LAPAROSCOPIC HERNIA REPAIR

The proponents of open hernia repair often put forward the performance of the operation under local anesthesia as an argument in its favor. Traditionally, laparoscopic hernia repair has been performed under general anesthesia. However, recent surgical literature shows several studies attesting to the safety and feasibility of spinal as well as epidural anesthesia for performance of TAPP as well as TEP.[12–14]

Schuricht *et al*[15] retrospectively compared the efficacy of epidural versus general anesthesia on length of stay, patient recovery and anesthetic-related complications in 167 patients undergoing 242 TEP procedures. Four patients required conversion of epidural anesthesia to general anesthesia because of inadequate sensory blockade. Sixty-seven patients underwent successful epidural anesthesia during the case, while 81 patients were managed with general anesthesia. The incidence of postoperative nausea was significantly lower (9%) in patients receiving epidural anesthesia as compared to general anesthesia (37%). Thirty patients (37%) in the general anesthesia group required analgesics for postoperative pain as compared to 13 (19.4%) in the epidural group. Thus, the authors advocated epidural anesthesia as an effective alternative to general anesthesia for the performance of outpatient TEP.

In the only reported study of its kind, Ferzli *et al* used local anesthesia for TEP in 10 patients (7 unilateral, 3 bilateral).[16] None of the cases required conversion to general anesthesia, but 4 required a small amount of intravenous sedation. The average lignocaine usage was 28 cc and the average operative time was 47 min. All patients were discharged within a few hours of the surgery and there were no complications. This study, although in a small number of patients, demonstrates the feasibility of TEP under local anesthesia. Larger studies, including prospective randomized comparisons between TEP and Lichtenstein repair under local anesthesia, are required to ascertain the place of this technique in laparoscopic hernia repair.

## COST IMPLICATIONS

Laparoscopic hernia repair is considered to be more expensive as disposable balloons are used to create extraperitoneal space during TEP, a much larger mesh as compared to open repair is used and fixation devices such as tacks are used for fixation of mesh. In the Western world, longer operative times resulting in increased theatre utilization charges is also a concern. Unfortunately, objective data analyzing the cost implications and cost-effectiveness of laparoscopic hernia repair are lacking.

Recently the Health Services Research Unit, Institute of Applied Health Sciences, University of Aberdeen, carried out an extensive review of literature to address whether laparoscopic methods were more effective and cost-effective than open mesh methods of inguinal hernia repair.[17] This review concluded that the laparoscopic repair was costlier than open mesh in all but 2 of the 14 studies. Laparoscopic repair was also found to be costlier to the health service than open repair, with an estimated extra cost of about £300–350 per patient. From the review of economic evaluations, the estimates of incremental cost per additional day at usual activities were between £86 and £130. Thus, where productivity costs were included in the analysis, they eliminated the cost differential between laparoscopic and open repair. In the review for the management of unilateral hernias, the open flat mesh was considered the least costly option but provided less quality adjusted life years than TEP or TAPP. TEP was preferred over TAPP as TEP was found to be less costly and more effective. For recurrent hernias, the data were sparse but extrapolation of data for primary repair was considered to provide the best available evidence. For management of symptomatic bilateral hernias, laparoscopic repair was considered to be more cost-effective as the operation time was reduced and differences in convalescence time are more marked for laparoscopic compared with open mesh repair. The study also addressed the issue of training costs. It was felt that for the National Health Service (NHS), increased use of laparoscopic repair would lead to an increased requirement for training which may be costly. During the training period, laparoscopic repair was thought to have higher costs (and hence was considered to be less cost-effective). Furthermore, the risk of serious complications was also thought to be higher during the learning curve.

From the Indian perspective, various factors come into play when analyzing the cost implications of laparoscopic repair of inguinal hernia. In most hospitals, except the larger corporate ones, the theatre time is charged on a per-case basis rather than by the hour. Thus, increase in the operating time, particularly during the learning curve, does not necessarily mean additional expense for the patient. If the surgeon were to adopt cost-containment strategies such as use of reusable laparoscopic instruments (which is more or less the norm in India) as against disposable ones, use of indigenous balloons devices rather than commercially available ones, sparing use of fixation devices and reliance on sutures for fixation of the mesh, the cost of the laparoscopic hernia repair should be comparable to the open repair. It is likely that many surgeons are already practicing these strategies and passing on the benefits of laparoscopic repair to their patients. It is imperative that high-volume centers in India undertake prospective studies to carefully document and analyze data related to the cost-effectiveness of laparoscopic hernia repair.

Repair of an incidentally detected contralateral hernia Laparoscopic repair of a unilateral inguinal hernia, particularly TAPP, provides the surgeon an opportunity to survey the contralateral side. The reported incidence of previously unsuspected contralateral defects (incidental defects) is 10% to 25%. Controversy exists about the routine repair of these hernias. Thumbe *et al* addressed this question in a small prospective randomized study.[18] Out of 32 consecutive men found to have incidental defects on the contralateral side during laparoscopic TAPP repair, 16 patients underwent simultaneous repair. In the remaining 16 patients, surgical repair was deferred. Subsequently, 5 consecutive patients found to have incidental defects were included in this group making the total number of patients with unrepaired defects 21. All the patients were followed up to detect any clinically demonstrable hernias. During a median follow-up of 15 months, demonstrable hernias developed in 6 patients (28.6%) in whom the hernias were not repaired at initial surgery. Thus this study demonstrated the benefits of simultaneous repair of an incidentally detected contralateral hernia. This argument would certainly hold true for the system in the UK, where this study was performed, as a ‘prophylactic’ repair would reduce the number of operations and hospital visits, thus reducing the cost to the NHS. However, in the Indian context, when the patient is often paying for the expenses out of his pocket, the additional cost implications of repairing an incidentally detected hernia should be kept in mind and perhaps also discussed with the patient preoperatively. Also, there appears to be an increasing and rather disturbing, trend in India that the third party payers do not readily pay for treatment undertaken for unsuspected pathology discovered during surgery. Thus, even when treating a patient who is insured, this aspect should be kept in mind when advocating repair of incidentally detected contralateral hernias.

## LAPAROSCOPIC REPAIR OF INCARCERATED INGUINAL HERNIA

Laparoscopic repair of incarcerated hernias is uncommon and remains controversial. Leibel *et al*[19] during a 6-year period repaired 220 incarcerated hernias, 194 of them by TAPP. The median operation time for TAPP was 55 min. A bowel resection was simultaneously undertaken in only 4 of the emergency cases (11.1%) and 2 of the chronically incarcerated cases (1.3%) in the TAPP group. The morbidity was 2.8% in the emergency group and 3.8% in the chronically incarcerated group, which was no different from the morbidity for TAPP used for treating reducible hernias. One recurrence was found 26 months after TAPP (0.5%). This study showed the feasibility and efficacy of TAPP in the treatment of incarcerated hernias.

More recently, Ferzli *et al*[20] retrospectively analyzed 11 cases of acutely incarcerated hernia in which TEP was attempted. Eight cases were completed successfully whereas 3 were converted to the open procedure. The mean operative time was 50 min and the mean length of hospital stay was 5.4 days. During a follow-up period of 9 to 69 months, there was no recurrence. There were two complications: one was an infected mesh that occurred in a case involving cecal injury. It was treated with continuous irrigation and salvaged. The other one was a midline wound infection after a small bowel resection for a strangulated hernia. The authors highlight the additional maneuvers that are necessary to place a releasing incision, at the same time safeguarding the hernial contents.

As there are only a handful of series addressing the issue of laparoscopic repair for incarcerated hernias and no prospective randomized trials comparing this approach to the conventional open repair have been conducted, meaningful inferences are difficult to draw. However, use of this approach remains in the realms of surgeons with extensive experience of elective laparoscopic hernia repair. The TAPP may be a better option in the setting of an incarcerated hernia as it provides better visualization and space for manipulation of viscera. A low threshold for conversion should always be maintained when attempting a laparoscopic repair for an incarcerated hernia.

The laparoscopic repair for groin hernias is still evolving. Several issues have been answered in the prospective studies carried out so far in the Western world. However, several more and larger studies are required from the developing world to address aspects unique to our circumstances. Finally, as Dr. Lloyd Nyhus, an authority in the field of hernia surgery, so eloquently put it several years ago: *‘The last chapter on the history of the groin anatomy and operative repair of hernia defects has not yet been written.’*
